# Missing from the Table: Role of the Environmental Public Health Community in Governmental Advisory Commissions Related to Marcellus Shale Drilling

**DOI:** 10.1289/ehp.1104594

**Published:** 2012-01-10

**Authors:** Bernard D. Goldstein, Jill Kriesky, Barbara Pavliakova

**Affiliations:** Graduate School of Public Health, University of Pittsburgh, Pittsburgh, Pennsylvania, USA

**Keywords:** drilling, hydrofracking, Marcellus Shale, natural gas, policy. *Environ Health Perspect* 120:483–486 (2012)

## Abstract

Background: The Marcellus Shale is a vast natural gas field underlying parts of Pennsylvania, New York, West Virginia, Virginia, and Maryland. Rapid development of this field has been enabled by advances in hydrofracking techniques that include injection of chemical and physical agents deep underground. Response to public concern about potential adverse environmental and health impacts has led to the formation of state and national advisory committees.

Objectives: We review the extent to which advisory committees formed in 2011 by President Obama and governors of the states of Maryland and Pennsylvania contain individuals with expertise pertinent to human environmental public health. We also analyze the extent to which human health issues are of concern to the public by reviewing presentations at the public meeting of the Secretary of Energy Advisory Board (SEAB) Natural Gas Subcommittee formed by the U.S. President’s directive.

Results: At a public hearing held by the SEAB Natural Gas Subcommittee 62.7% of those not in favor of drilling mentioned health issues. Although public health is specified to be a concern in the executive orders forming these three advisory committees, we could identify no individuals with health expertise among the 52 members of the Pennsylvania Governor’s Marcellus Shale Advisory Commission, the Maryland Marcellus Shale Safe Drilling Initiative Advisory Commission, or the SEAB Natural Gas Subcommittee.

Conclusions: Despite recognition of the environmental public health concerns related to drilling in the Marcellus Shale, neither state nor national advisory committees selected to respond to these concerns contained recognizable environmental public health expertise.

The development of hydrofracking technology has led to rapid growth in drilling for oil and natural gas in the United States and globally. Public concern about potential environmental and public health consequences has led to the formation of governmental advisory committees that are looking at the risks and consequences of the drilling activity. In 2011, President Barack Obama and the governors of Pennsylvania and Maryland independently established commissions to provide advice about a broad range of issues related to drilling for natural gas.

The Marcellus Shale is a rich natural gas field, said to be the second largest in the world, that extends under much of Pennsylvania, New York, and West Virginia and parts of Maryland, Ohio, Virginia, Kentucky, and Tennessee (Considine et al. 2010). The technology that permits access to natural gas in the Marcellus Shale includes drilling first vertically to the shale level and then horizontally within the shale (Arthur et al. 2009; Pennsylvania Department of Environmental Protection 2011). Holes are then cut in the horizontal pipe, followed by injection of high volumes of hydraulic fracturing fluids (primarily water and sand plus chemical additives) to break open the shale layers and maintain gas flow (for a compendium of links to research and data on the Marcellus Shale, see FracTracker 2011).

The amount of water used is in the range of a million gallons per well injected over perhaps days to a few weeks (New York City Department of Environmental Protection 2009), and the level of chemicals additives is in the range of 0.5–2.0% [U.S. Department of Energy (DOE) 2009]. About 30–70% of the fracking fluid returns to the surface and must be discharged somewhere (DOE 2009). Specific chemical and physical agents used in the fracking mixture to increase the release and flow of the fossil fuel and prevent microbial growth, corrosion, and scale formation vary by company and by location. Lists of these additives have been published (U.S. House of Representatives 2011), and some companies have been cooperative in revealing additives used (FracFocus Chemical Disclosure Registry 2011; Range Resources 2010), but the lack of complete information complicates interpretation of public complaints about health impacts. Secrecy itself may heighten public concern, as appeared to occur as a result of the high-volume use of a dispersant with an unknown component during the *Deepwater Horizon* disaster (Goldstein et al. 2011). Other unknowns include whether fracking chemicals react with other compounds to produce new chemicals, and whether naturally occurring agents such as arsenic, bromine, and radioactive compounds may be displaced into waste fracking fluids or groundwater (Goldstein 2011). Concerns about potential endocrine-disrupting effects of fracking chemicals have been raised (Finkel and Law 2011) and the potential health impacts of oil and gas development have been reviewed (Witter et al. 2008).

In the context of environmental and public health concerns, we reviewed the organizational aspects of three of these advisory committees related to drilling for natural gas in the Marcellus Shale and nationally. We also analyzed the extent to which human health issues are of concern to the public by reviewing presentations at the public meeting of the Secretary of Energy Advisory Board (SEAB) Natural Gas Subcommittee.

## Materials and Methods

We reviewed the charge and the composition of three advisory committees formally established in 2011 to review drilling for natural gas. Two were established by governors, one Republican (Pennsylvania Governor Tom Corbett) and one Democrat (Maryland Governor Martin O’Malley), and the third by President Obama, also a Democrat. We classified individual committee members according to employment or participation in government, academia, environmental groups, civil society groups, or industry, based on their current positions. Possible health expertise was determined through review of biographies available as part of the official record of their appointment, review of information available on their organization’s website, or a web search via Google.

For this study, we distinguished between environmental public health, which focuses on the human consequences of an activity affecting the environment, and environmental health, which we define more broadly as alterations of the environment. We recognize that this is an arbitrary distinction, and that there is a continuum between environmental and human health. By identifying a committee member as having environmental public health expertise, we mean that this individual has experience in evaluation of or response to the direct toxicological effects of chemical and physical agents on human health or indirect effects such as psychosocial stresses, highway safety, and drug and alcohol abuse. For the purposes of this study, we have extended this definition to include medical and health professionals who could be presumed to have some health background related to environmental health, however minimal (e.g., physicians, nurses, pharmacists, psychologists).

Public response was assessed by review of presentations to the 13 June 2011 federal SEAB Natural Gas Subcommittee meeting held in Washington, Pennsylvania, the one public meeting of the subcommittee in the Marcellus Shale area. The meeting was widely advertised and well attended both by supporters and by opponents of Marcellus Shale drilling, many of whom were from surrounding states. Because of the large number of speakers, each was limited to two minutes. Two of us (B.P. and J.K.) independently reviewed the video of these presentations (SEAB Natural Gas Subcommittee 2011) to develop a list of codes summarizing the main points covered by each speaker and categorize speakers as either supporters (51) or opponents (59) of shale gas drilling. The responses of the opponents were further categorized into a variety of subheadings. Differences between the two raters were reconciled before analysis.

## Results

*Review of the executive orders.* U.S. federal government. The federal review of fracking issues was requested in President Obama’s “Blueprint for a Secure Energy Future” (Obama 2011) that states:

To provide recommendations from a range of independent experts, the Secretary of Energy, in consultation with the U.S. EPA Administrator and Secretary of Interior, should task the Secretary of Energy Advisory Board (SEAB) with establishing a subcommittee to examine fracking issues. The subcommittee will . . . include leaders from industry, the environmental community, and states. The subcommittee will work to identify . . . any immediate steps that can be taken to improve the safety and environmental performance of fracking and to develop . . . consensus recommended advice to the agencies on practices for shale extraction to ensure the protection of public health and the environment.

Note that this executive order gives leadership to the DOE in consultation with the Department of Interior and U.S. Environmental Protection Agency (EPA). Although this statement culminates with the charge to ensure the protection of public health and the environment, the U.S. Department of Health and Human Services (DHHS), despite its environmental health components [National Institute of Environmental Health Sciences (NIEHS), Centers for Disease Control and Prevention/Agency for Toxic Substances and Disease Registry], is not included.

Pennsylvania. In Pennsylvania, the newly elected Governor, Tom Corbett, had stressed the importance of the Marcellus Shale to economic development during his campaign. Early in his administration, in March 2011, he appointed a 31-member Governor’s Marcellus Shale Advisory Commission. The executive order establishing the commission states:

The Commonwealth takes seriously its responsibility to ensure the development of gas in a manner that protects the environment and safeguards the health and welfare of its citizens. (Corbett 2011)

Four work groups were designated by the commission, including one on public health, safety, and environmental protection. They were charged with the following task:

Consideration of additional measures necessary to ensure the protection of the Commonwealth’s environment and natural resources and the enhancement of public health and safety. (Governor’s Marcellus Shale Advisory Commission 2011b)

Maryland. In July 2011, Governor Martin O’Malley of Maryland appointed a 14-member commission. The Governor’s Executive Order specifically states:

Purpose. The Marcellus Shale Safe Drilling Initiative will assist State policymakers and regulators in determining whether and how gas production from the Marcellus Shale in Maryland can be accomplished without unacceptable risks of adverse impacts to public health, safety, the environment and natural resources. (O’Malley and McDonough 2011)

In summary, public health and the environment are featured in the rationale for the formation of all three committees formed to give advice on Marcellus Shale drilling.

*Composition of the advisory committees.* The total number of appointments to these three advisory committees was 52, consisting of 51 individuals. One individual, Jeffrey Kupfer, an energy company executive who was the former DOE Deputy Secretary, was chosen as a member of both state advisory committees.

The SEAB Natural Gas Subcommittee consisted of seven members (DOE 2011): three in academia, three in industry, and one in an environmental group (Fred Krupp, President of the Environmental Defense Fund). The chair of the SEAB subcommittee, John Deutsch, is the former Chairman of the Department of Chemistry, Dean of Science, and Provost at Massachusetts Institute of Technology. Other members are Stephen Holditch, head of the Department of Petroleum Engineering at Texas A&M University, and D. Mark Zoback, professor of geophysics at Stanford University. Several of the subcommittee members have experience in more than one sector. For example, Deutsch is a former Deputy Secretary of Defense and the former head of the Central Intelligence Agency and is on the board of various energy-related companies. Kathleen McGinty of Weston Corporation was previously head of the White House Council on Environmental Quality under President Bill Clinton and was Pennsylvania Secretary of the Environment under its previous governor.

The Pennsylvania Governor’s Marcellus Shale Advisory Commission has 31 members: 10 from government, 1 from academia, 4 from environmental groups, 5 from civil society groups, and 11 from industry (Governor’s Marcellus Shale Advisory Commission 2011a). It was chaired by the lieutenant governor. The one academic, Terry Engelder, is a professor of geoscience at the Pennsylvania State University whose research on the extent and availability of natural gas in the Marcellus Shale has been recognized as central to its current rapid development.

The Maryland Marcellus Shale Safe Drilling Initiative Advisory Commission has 14 members: 6 from government, 1 academic who chairs the commission (David Vanko, a geologist and current Dean of the College of Science and Mathematics at Towson University), 3 from environmental groups, 2 from civil society groups, and 2 from industry (Maryland Department of the Environment 2011).

*Environmental public health or other health expertise.* Our review of the background of all 51 members of the three advisory committees provided no evidence that any member had expertise in the human health aspects of environmental health or experience in health or health care. Based on the available information, we were unable to identify any public health personnel, physicians, nurses, pharmacists, dentists, or others with a health background on the three advisory committees.

*Evaluation of public concerns.* Categorization of the 110 public comments at the SEAB Natural Gas Subcommittee meeting in Washington, Pennsylvania, revealed 51 speakers favorable to shale gas drilling and 59 opposed (Natural Gas Subcommittee 2011). Among the opposed, the major concerns discussed included the negative effects of Marcellus Shale drilling on the environment expressed by 46 speakers (78%), concern regarding the safety and/or regulation of the natural gas drilling industry expressed by 41 (69.5%), and concern for residents’ health expressed by 37 (62.7%) (Table 1). Other concerns ranged from general effects on air, water, and ecosystems to concerns about the potential for negative health effects on humans. Of those opposed, 12 (20.3%) attributed a direct negative health impact on themselves, a family member, or a friend to Marcellus Shale drilling. Almost a quarter of the speakers opposed to drilling expressed concerns regarding the make-up of the committee, including the potential for bias toward industry interests and a lack of expertise among committee members regarding their specific concerns.

**Table 1:** Concerns raised by opponents (*n* = 59) of Marcellus Shale drilling at the Washington, Pennsylvania, public meeting with the SEAB Natural Gas Subcommittee.

Concern	*n* (%)
Environmental concerns	46 (78.0)
Safety and regulation of industry	41 (69.5)
Negative effects on water	39 (66.1)
General health concerns	37 (62.7)
Negative effects on air	23 (39.0)
Chemicals in water	22 (37.3)
Bias, conflict of interest, or lack of expertise in desired subject area by members of the committee	14 (23.7)
Health problem in family member attributed to drilling	12 (20.3)
Personal legal rights have been infringed upon by companies	8 (13.6)
Export of domestic natural gas resources	6 (10.2)
Depreciation in property values	4 (6.8)


## Discussion

Recent technological and operational improvements in extracting natural gas resources, particularly shale gas, have increased gas drilling activities nationally and led to significantly higher natural gas production estimates for decades to come (Considine et al. 2010). The potential for adverse environmental public health consequences has been recognized by the public, who are concerned, and by members of the government, who have asked for advice. However, despite these stated concerns for potential public health impacts from Marcellus Shale activities, none of the three recently formed advisory bodies include any recognizable expertise in assessing environmental impacts on human health, nor have they invited participation from state or federal agencies with direct public health responsibilities.

This is not the only recent instance in which environmental public health expertise has not been at the table despite obvious human health implications of an environmental issue. President Obama’s seven-member National Commission on the BP *Deepwater Horizon* Oil Spill and Offshore Drilling was co-chaired by former U.S. EPA administrator William Reilly and Senator Bob Graham and has one member, Donald Boesch, who is a professor of marine science and has extensive experience in ecosystem research. However, no members of this committee have a background in environmental public health. Other recent advisory committee reports on natural gas extraction include that of the National Petroleum Council, whose membership primarily is from the petroleum industry but also includes government personnel and university faculty. Its extensive report of impacts on wildlife habitat and on the environmental footprint of oil exploration and operations includes only an occasional reference to human health (National Petroleum Council 2011).

We can only conjecture about the reasons that environmental public health experts or organizations have not been included in advisory bodies related to Marcellus Shale activities. It is unlikely that the failure to include environmental public health expertise is due to lack of recognition that there is reason to be concerned about human health risks. Such concern is clearly stated by the two state governors and by President Obama in establishing their advisory committees. Nor can it be a lack of awareness of the well-publicized public concerns about the potential health impacts of Marcellus Shale activities, which have been forcefully described by the public in numerous hearings.

In Pennsylvania, the absence of the Department of Health or of any public health expertise in the 31-member Governor’s Marcellus Shale Advisory Commission could reflect the relative weakness of that state’s public health infrastructure. Although having expert health departments at the state level and in some localities, Pennsylvania ranked last nationally in a survey sponsored by the Health Resources and Services Administration on the size of the public health workforce in each state (37 per 100,000, vs. the national mean of 138 per 100,000) (Gebbie et al. 2000; Potter 2008). However, Maryland did relatively well in this regard (304 per 100,000) and ranked first in its region, which includes Pennsylvania.

University-based expertise is well represented in the federal SEAB subcommittee, with three of its seven members being respected academics, but academia is not particularly well represented in the two state advisory committees. In Pennsylvania, only one of the 31 members has a direct university affiliation. Maryland also has only one of its 14 members from academia, although in this case it is the committee chair. None of the three committees includes members with academic expertise in health science or ecosystem sciences; that is, none of the five academic members has expertise in biological systems or human health.

Ecosystem concerns are represented in these three advisory committees through the presence of leaders of environmental organizations that are particularly involved in ecosystem health, such as the Nature Conservancy, Trout Unlimited, Savage River Watershed Association, Pennsylvania Environmental Council, Western Pennsylvania Conservancy, and Chesapeake Bay Foundation. But there is no representation from organizations known primarily for their concern about human health and the environment. Some of the environmental organizations whose leaders are members of the advisory committees, such as the Environmental Defense Fund, have been involved with human health issues as well as more general environmental preservation. But none of the advisory committee members has personal expertise in human health.

The failure to choose academics that have expertise in human health issues and the environment is not because of lack of such expertise. Both Maryland and Pennsylvania have reasonably robust academic public health infrastructures, with each having two accredited schools of public health, and Maryland having three and Pennsylvania five accredited programs of public health. All four schools of public health have formal departments in the field of environmental health, and accreditation as a program requires at least sufficient faculty to teach a core course in environmental health. Both states do well in another indicator of academic expertise in environmental health sciences, that of funding from the NIEHS. In fiscal year 2010, Maryland received 4.7% of total NIEHS funding and Pennsylvania 4.3%; the states have 1.9% and 4.1% of the total U.S. population, respectively (DHHS 2011).

Political and bureaucratic issues deserve further consideration. President Obama gave the lead on Marcellus Shale to the Secretary of Energy in consultation with the Department of Interior and the U.S. EPA. The rationale for not including the DHHS is not clear. Arguably, the DOE does have some health expertise, because it has various organizational structures dealing with health and safety related to energy or to the cleanup of atomic materials production sites. Although conceivable, we do not believe that the authorities fail to recognize the difference between environmental and public health expertise, particularly because in each case the executive orders separately specify environment and health.

One can argue that the U.S. EPA, which at its formation included components moved from the U.S. Public Health Service, does have public health responsibilities (Goldstein 1988; Johnson 2010). Evidence that the U.S. EPA does take its public health responsibilities seriously includes a recent reorganization to include an Environmental Public Health division (U.S. EPA 2012). Lisa Jackson, the Administrator of the U.S. EPA, in speaking about sustainability, often uses classic public health language by pointing out that sustainability is similar to pursuing wellness instead of treating disease (Jackson 2010).

Congress provided the U.S. EPA’s Office of Research and Development with funding specifically to look at groundwater contamination from Marcellus Shale hydrofracturing (U.S. EPA 2011). This appropriation did not allow studies of the potential environmental or human toxicity of fracking compounds or of the potential for air pollution—although it is not clear why the U.S. EPA does not use other funding for such research. This at least raises the possibility that political leadership does not want research on human health because of concerns that equivocal or positive findings might inhibit economic development or offend major industries. Environmental public health experts may be seen as more likely to raise problems than to find solutions.

To explain the absence of environmental public health expertise in governmental advisory processes related to the Marcellus Shale, we are left with the distinct possibility that it is the fault of the environmental public health community. We have not worked as hard or as effectively as we could or should with local, state, or federal governmental organizations responsible for making decisions on environmental matters. These governmental organizations extend well beyond state departments of health or the federal units that are part of the DHHS. Simply being able to provide the science needed for effective environmental decision making is not sufficient if our science does not inform decisions that are made. A proactive approach to working with the broad range of federal and state agencies involved in environmental decision making, and with the public, including providing economic analysis related to potential positive and negative health impacts, is central to converting our knowledge to protection of human health and the environment (Hearne 2008; Longest and Huber 2010; Rutkow et al. 2009)

## Conclusions

Environmental public health is not yet at the table in governmental advisory processes related to drilling in the Marcellus Shale. The explanation for the lack of involvement of the environmental public health community does not appear to be a failure to recognize the importance of public health to this issue by the president or the governors, nor is it a lack of public concern. Expertise in the impact of environmental factors on public health is also readily available.

Political concern that evaluation of the potential environmental public health consequences of shale gas drilling may find a problem that slows down the rush to develop the Marcellus Shale may be an important factor. However, we believe the most likely major cause is the failure of a relatively robust community of environmental public health experts to adequately project this expertise into the state and national debates about developing natural resources.

Larry Gordon (1990) has noted the lack of involvement of environmental public health in key environmental issues. He emphasizes the importance of increasing the numbers and the training of the environmental health workforce. More than two decades later, review of the advisory processes developed to make recommendations concerning natural gas suggests that we need to go beyond standard infrastructure issues to consider how best to project environmental public health concerns at the local, state, and national levels.
